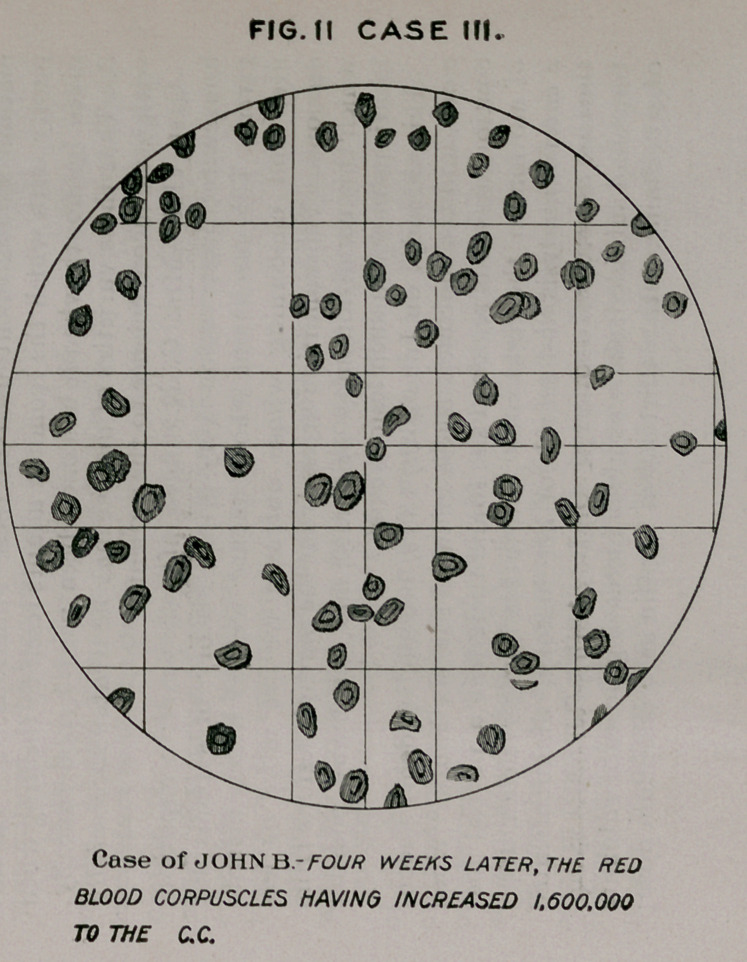# The Gold Combinations as Alternatives*Read before the Mississippi Valley Medical Association, at its twenty-first annual meeting.

**Published:** 1895-12

**Authors:** Thomas Hunt Stucky

**Affiliations:** Professor of Theory and Practice, and Clinical Medicine, Hospital College of Medicine, Louisville, Kentucky


### Selections and Abstracts.

[*New York Medical Journal.*]

### THE GOLD COMBINATIONS AS ALTERATIVES.*

By THOMAS HUNT STUCKY, M. D., Ph. D.

Professor of Theory and Practice, and Clinical Medicine, Hospital College of Medicine, Louisville, Kentucky.

At a meeting of the Medico-Chirurgical Society, April 15th, 1895, I had the pleasure of exhibiting a series of cases which had been taking the preparations of gold and arsenic, known to the profession as arsenauro and mercauro. I was under the impression at the time that the good effect claimed was produced in three ways:

1. By stimulation of the secreting glands of the stomach.
2. By the probable alterative effect upon those secretions.
3. That a local antiseptic influence was exerted.

Having continued my experiments in a vast variety of cases, both acute and chronic, and with varied effects and such unexpected results, I concluded at the first opportunity, if possible, to learn wherein and how these combinations exerted their peculiar and, in many respects, wonderful influence. This opportunity was afforded during my hospital service, which commenced April 15th last, or about four months ago. At this time of the year, the public wards, as a rule, are free from acute diseases, and the patients were mostly of phthisis, Bright's disease in its various stages, chronic hepatic troubles, and convalescents. I made it a rule with all these cases to withdraw all medicines, except combinations of gold and arsenic. I have selected, from a series of cases, some four or five, which, with your permission, will be read:

Case I.—J. H., white, aged sixty years; family history good; previous to April, 1894, in good health; normal weight, one hundred and forty-five pounds; present, one hundred and four. Although very feeble has not taken to bed. On physical ex-

*Read before the Mississippi Valley Medical Association, at its twenty-first annual meeting.

amination, the infraclavicular region of the right side was seen to be flattened, with diminished resonance and numerous moist rales, considerable cough, and mucopurulent expectoration, which contains the tubercle bacilli; has had loss in weight under continuous treatment during the previous six months; temperature ranging from 99.5 degrees to 102 degrees Fahrenheit; pulse 96 to 110 a minute. On April 22d, 1895, eight drops of the mercuric bromide of gold and arsenic were given, hypodermically, every four hours, this treatment being continued for six weeks. No deleterious results were noticed; on the contrary, he is decidedly better; physical condition, color, bodily strength and appetite improved, being now employed as a waiter. The blood counts made at the beginning and the end of the course illustrated well the improvement which had taken place; they are as follows:

April 22d.—Corpuscles, 3,800,000 to the cubic millimeter; hæmoglobin, fifty-five per cent.

June 19th.—The red corpuscles had increased to 5,378,000 and the hæmoglobin to eighty-two per cent. At this time cough and expectoration have disappeared and the moist rales no longer heard; temperature normal; pulse about ninety a minute; deficiency in resonance and expansion remain; tubercle bacilli not found.

The two points of interest in this case are, first, the increase in the number of red corpuscles; second, and more important, the increase in quality of the corpuscles as demonstrated in the increase of hæmoglobin. The next case is of considerable interest.

CASE II.—F. P., aged sixty-five, history of dissipation, admitted November, 1894; much jaundiced; pain in right hypochondriac region; pain and jaundice gradually disappeared, leaving him much emaciated; anorexia; bowels constipated; diagnosis, cirrhosis of liver. Urine shows no marked deviation from health. Blood contains many small and large red cells, the red corpuscles numbering 3,253,000; hæmoglobin, fifty-two per cent. Treatment: arsenauro, eight drops every four hours, hypodermically, commencing April 22d.

May 5th.—Patient appears to be stronger, remaining out of bed and not requiring purgatives, as formerly. Examination of the blood at this time shows 4,300,000 red corpuscles to the cubic millimeter; hæmoglobin, sixty-five per cent.

31st.—While still using the gold combinations, there was a diminution in the number of red corpuscles to 3,850,000, and in hæmoglobin to sixty per cent.

June 19th.—Patient seems to be in fairly good condition. Duirng the past week he suffered from abdominal pain, diarrhœa following this attack. Treatment continued.

20th.—Examination shows 4,650,000 red corpuscles; hæmoglobin seventy-five per cent.

While there have been fluctuations in the condition of the patient he is, after all, much better as regards appetite and bodily vigor.

CASE III.—John B., teamster. Notes of this case began in 1893. He then had flattening, especially of the right side, diminished resonance, pain in supraclavicular region; nocturnal cough; mucopurulent expectoration. The tubercle bacilli could not be found, and many slides examined during the following two years failed to reveal their presence.

Changes in the physical signs have been slow; the area of fullness has extended to the right side, the heart is drawn to the right. The left lung presents the same signs as the right, but not so pronounced; he has constant fever, the evening rise usually 101 degrees and not uncommonly reaching 103 degrees. The treatment in this case has been varied, including strychnine, cod liver oil, hypophosphites, and the ferruginous combinations. There had been no improvement in his general condition for three months before the administration of mercauro. He remained in bed, appetite poor, anæmic, bowels constipated.

No examination of the blood had been made prior to April 20th, the day he was placed upon mercauro. At that time, the blood corpuscles were 3,400,000; hæmoglobin, sixty-five per cent. About ten days after this treatment was instituted there occurred a very remarkable increase in the appetite, with the complete disappearance of constipation. Four weeks later, after having been in the hospital for two years, he was sufficiently recovered to leave. The corpuscular count was normal; hæmoglobin, eighty per cent., and he had gained ten pounds in weight.

CASE IV.—John H., aged sixty-five years, habits temperate; this patient was one of the few survivors of the pneumonia epidemic of last winter. He had a mild form of the disease, but it left him extremely feeble. Had been treated with Fowler's and Donovan's solution of arsenic, with the bitter tonics, malt, and stimulants, from March 14th to April 20th. At the end of this time, he was scarcely able to sit in an easy chair, could not stand alone, very pale, pulse feeble and intermittent, bowels constipated, complete anorexia. On physical examination, there were pronounced dullness, harsh breathing, and moist rales over lower lobe of the right lung, the upper lobe of the left being clear.

April 22d.—Placed upon arsenauro, hypodermically, every four hours.

May 3d.—The following is taken from bedside notes:

"Patient eats a great deal, complexion good, walks about the ward, lung almost clear, no cough, no expectoration." The rapid improvement continued, and the patient was dismissed May 20th, able to work at his trade. It should be noted that after five weeks' use of solutions of arsenic, bitter tonics, and alcoholic stimulants, he had 4,000,000 red corpuscles to the cubic millimeter and hæmoglobin, forty-seven per cent. Under administration of bromide of gold and arsenic, the hæmoglobin increased to eighty-five per cent, and the red corpuscles to normal.

CASE V.—Jacob H., aged sixty years. The patient, a Russian Jew, is deaf and understands very little English. Examined April 21st, 1895. Heart sounds normal; urine presents no striking abnormity. Chronic bronchitis; chalky deposits in different joints, particularly carpo-metacarpal, causing the usual grating sound when manipulated; knee and ankle joints painful—so much that he is unable to walk; no fever; anæmic. The blood count showed 4,000,000 red corpuscles; sixty per cent. hæmoglobin; ten drops of mercauro ordered [illegible]ypodermically, every four hours.

May 10th.—Lungs clear, cough and expectorations ceased, walks everywhere. Discharged, cured of cough and pain May 28th, corpuscular count showing 5,450,000 red corpuscles; hæmoglobin, eighty-five per cent., or an increase, in one month, of twenty-five per cent.

CASE VI.—Came under my treatment January 16th, 1895. Mrs. W., aged thirty-seven years, preservation good, temperament nervous, being intelligent and cultured. She has been a morphine habitue for the past six years; this habit was induced by small quantities being given to alleviate pain, which she maintained originated from a lacerated cervix uteri. This laceration had been successfully repaired, but the desire for morphine still existed, and several futile attempts to rid her of the noxious habit had been made. When she applied for treatment her daily amount was about fifteen grains, which was taken by mouth. The method to be pursued in treatment, judging from the condition of the patient, was to decrease the amount taken by the fractional method of giving half the quantity received the preceding day. To combat the nervous disturbances anticipated by the withdrawal of the morphine, two drachms of the fluid extract of Jamaica dogwood and half an ounce of wine of coca were ordered every four hours. The result of this was not as expected, since on January 19th she was receiving three grains a day; the nervous disturbances were so great that it seemed unsafe to continue the treatment. Her temperature, at this time, was 97 degrees Fahrenheit. Pulse rate 110, and respiration 26, respectively, per minute. Her appetite was much lessened, and was replaced instead by nausea; a serious diarrhœa also existed. The treatment, however, was carefully continued. On January 21st, when only one grain a day was being taken, her chart showed that the loss of appetite, nausea, diarrhœa had become anorexial, vomiting and purging accompanied by continuous muscular vibrations. This resulted in an increase of the morphine to three grains a day, with the dogwood and coca discontinued. At this date liquor auri, arsenii, et hydrargyri bromidi (Barclay), ten drops every four hours, hypodermically, was ordered, with no decrease in the amount of morphine taken.

January 23d.—The alarming symptoms still persist.

24th.—Oscillations throughout muscular system are much less marked, with some intermissions; diarrhœa not so severe.

25th.—Only six stools during the day; vomiting has ceased; some hot milk was retained in the stomach.

FIG. I CASE II.

A. MICROCYTE. - B. MACROCYTE. - C. LEUCOCYTE.

Case of F.P. – CIRRHOSIS OF LIVER WITH JAUNDICE, BLOOD CHANGES OF SEVERE SECONDARY ANÆMIA.

FIG. II CASE II.

SHOWS INFLUENCE OF ARSENAURO UPON THIS CONDITION BY THE GREAT INCREASE IN NUMBER, SIZE AND QUALITY OF RED BLOOD CORPUSCLES

26th.—The patient slept well during the night; has had no stools; ate some solid food, trembling almost disappeared; no morphine had been given during the day and no desire for same.

The patient continued under treatment, and improved with careful watching. On February 8th the solution of bromide of gold, arsenic and mercury was ordered to be decreased one drop a day. She was discharged April 10th, cured permanently.

CASE VII.—On February 4th, 1895, Mr. H. came under my observation during the course of Case VI. Age thirty-two years; preservation good; color exceedingly pale. This man presented the same malady as the patient in Case VI., having been a morphine eater during the past four years. Several futile attempts toward a withdrawal of the drug had been made, using various methods of treatment. The method of treatment in this case was materially different from that advocated in Case VI., since his daily amount of morphine, which was twenty grains hypodermically, was diminished less rapidly, and at the same time the diminution was supplemented by increasing doses of nitrate of strychnine, commencing with a thirtieth of a grain increased to a fifteenth, this being given hypodermically. The hypodermic syringe had always been used by him, resulting in a mutilated cutaneous surface by needle puncture. In order to preserve this surface as much as possible, the daily amount, twenty grains, was ordered by mouth.

This apparently had no effect in satiating the demand, which required the syringe the following day; his anæmic appearance suggested the examination of his blood, which was made without further delay. The corpuscular enumeration amounted to 4,756,000, which was practically normal. The relative proportion of the white to the red was one to six hundred.

The corpuscular elements were, however, far below normal, since his hæmoglobin was only thirty-seven per cent. normal. This, we concluded, gave origin to his extreme pallor. The treatment had been in progress only four days when the patient became very much discouraged, at the same time abandoning the attempt. This loss of moral courage was

FIG. I CASE III.

Case of JOHN B.- *CHRONIC PULMONARY TUBERCULOSIS, SHOWING MARKED DECREASE IN THE RED BLOOD CORPUSCLES.*

FIG. II CASE III.

Case of JOHN B.- *FOUR WEEKS LATER, THE RED BLOOD CORPUSCLES HAVING INCREASED 1,600,000 TO THE C.C.*

counterbalanced by a complete saturation of the system with morphine. This induced him again to apply for treatment. Instead of continuing the treatment on the same principle as before mentioned, the strychnine solution was abandoned; mercauro, eight drops every two hours, was given during the first two days, with the same quantity every six hours during the following seven days. The morphine was diminished a grain a day. At the end of ten days, his condition was very good, having had no marked nervous disturbances, little loss of appetite, and no diarrhœa. The mercauro, on February 18th, was reduced to six drops every four hours, morphine being discontinued. On March 1st, no morphine was being given, all desire for its effects having disappeared; the mercauro was ordered given by mouth. His color was much improved; his appetite for morphine no longer existed; his movements and speech had become composed. He was discharged April 1st, with a satisfactory result. The red corpuscles numbered 4,600,000 to the cubic millimeter.

These two cases are interesting to us from several points of view: 1. They show the comparative values of several methods of treatment used in these afflictions. 2. The impunity with which the economy adapts itself to the drug when given by mouth when it has once been used hypodermically. 3. That these varieties of disease may be treated successfully with little inconvenience to the patient.

CASE VIII.—Mrs. C. N., aged fifty-six years; occupation, housewife; preservation very good; history of syphilis not given. This case is one whose nature we find widely distributed and concerning whose outcome we are more or less anxious. This condition arises from the multiple lesions from which this condition may originate, and the many possible locations in which such lesions exist.

This old lady, on June 26th, 1894, became suddenly unconcious, and the unconciousness endured for six hours. When conciousness was regained she found there was a partial loss of motion on the right side. The attending physician, after a careful analysis and search of her history, diagnosticated the case as cerebral apoplexy. On July 13th, after acute symptoms had subsided, she was given increasing doses of sulphate of strychnine, with a thirtieth of a grain as a minimum dose; the doses were given three times a day in conjunction with electricity.

This was continued until January 2d, with apparent but not positive results, since only partial sensation, with no motion, had returned. At this time, she applied to me for treatment. Her muscles, on the affected side, were remarkably atrophied, with a tendency to secondary contraction. At this time, she was receiving half a grain of strychnine three times a day; this, with the electricity, was discontinued, liquor auri, arsenii et hydrargyri bromidi (Barclay), six drops every four hours, hypodermically, being used. Passive muscular action daily was advised. A comparison of the right and left muscular systems, respectively, was also ascertained at this time. Around the right deltoid region measured twelve inches; left, thirteen inches; right bicipital region, eleven inches; left, twelve inches and a quarter; right bicipital, during flexion, eleven inches and a half; left, fourteen inches and a quarter; right middle third of thigh, twenty inches and a quarter; left, twenty-two inches and a quarter; right calf, twelve inches; left, thirteen inches and a quarter. The treatment suggested was faithfully executed. In a few weeks, improvement was noticed, which continued.

On April 22d, another examination of her condition was made. At this time, she could feel distinctly whatever came in contact with the parts affected. By means of a dragging motion, she was able to go from one place in the room to another. Extension of the forearm and the fingers could be almost complete, while the flexor muscles registered to the point twenty on the manometer.

Seeing the past improvement, the gold was still continued, with the expectation of a near approach to recovery.

On May 20th, her entire arm could be extended to the plane of the shoulder; extension was very good; the flexor muscles of the hand had recuperated so that they registered thirty points more in strength on the manometer; walking was accomplished readily with the aid of a cane.

We see here a case, apparently hopeless, having reached a point in recovery, providing the patient with power to do housewife duties. I selected these few cases out of a large number to demonstrate, in my judgment conclusively, that by the combination of gold and arsenic, we have an agent acting as neither of the minerals when administered separately; or, in other words, we have an entirely new agent in so far as therapeutic action is concerned.

It will be worth our while to look into the chemical differences between the chloride of gold and sodium (salted chloride of gold) and the bromide of gold and arsenic (arsenauro) with reference to its therapeutic action and subsequent elimination.

While not attempting to solve a question which has puzzled experienced men, a few remarks regarding the chemical differences of these agents may furnish a groundwork for an original theory.

1. The chloride of gold and sodium of commerce, so-called, is not such in fact, but merely chloride of gold mixed with chloride of sodium; therefore, for any chemical purposes, chloride of gold only need be considered.

2. Chloride of gold is an extremely unstable compound, its identity being readily destroyed by light or air, while the addition of the least amount of organic matter will almost instantly convert it into albuminate, which, upon contact with the mucous membrane or skin surface (the albumin thus formed), is etxremely difficult of solution.

3. Gold bromide, even without the addition of the other material, is a more stable salt, is less sensitive to light, etc., and, when in combination with bromide of arsenic in aqueous solution, as found in arsenauro and mercauro, this property of stability is increased to a seemingly very great extent.

4. This change in its attitude with reference to outside influences, from a chemical standpoint, may account for its altered therapeutic properties, and this may be said not only as regards the changes due to the combined therapeutic properties of the combination of gold and arsenic, but with reference solely to the probable modified or intensified quality, which appears to be a changed therapeutic equivalent in the gold itself.

5. As to what I conceive to be the reason of the changed or intensified therapeutic quality of gold in arsenauro, etc. [illegible]he arsenic added to this solution appears to have rendered the gold more tenacious of its dissolved condition, thus permitting it to be taken unaltered into the circulation.

The finding of gold in the urine after the administration of these solutions, would appear to confirm this view.

Taking the formulæ of the two preparations, Fowler's solution would appear to be about thirteen times as strong in arsenic.

One would naturally expect to observe a corresponding therapeutic potency; such, however, is not the case.

Fowler's solution often causes stomach disturbances, and often exhibits suddenly what appear to be cumulative effects.

Such is not true of arsenauro, even though the full therapeutic effect of arsenauro is being obtained.

Fowler's solution is probably decomposed, upon entering the stomach, into chloride of potassium and arsenious acid; at any rate, after poisoning with Fowler's solution in quantities, arsenious acid has been found in the folds of the mucous membrane, enough having been redissolved or taken up before precipitation to kill. Arsenious acid is with difficulty soluble in the complex organic contents of the stomach.

These difficulties may be due to conditions in the metals themselves; due to the combination, or to a possible new salt thus formed. Certainly the gold found in the combination is more stable and tenacious of its dissolved condition, and certainly the arsenic seems to be more readily absorbed, and to exert its therapeutic effect much more constantly and with a much smaller dose, and to be entirely free from that quality common to all other arsenical preparations, stomachic disturbance. As said before, this may be due to the combination of the two alterative tonics, or of a changed therapeutic equivalent in one or both metals by their chemical action on each other. My experience up to April 1st, 1894, had been in the administration of these products entirely by the mouth. Numerous writers, within the past year, reported some very unusual results obtained by their use in indiscriminate cases, without any regard to any direct line of therapeutic application; or, in other words, that the therapy of the drug was not known. It seemed to be a sort of stopping-off drug, and when everything else failed, a solution of the gold was tried.

It was with this idea in view, and the knowledge, or rather lack of knowledge, that led to these experiments. I believe that in the action of the combination of bromide of gold and arsenic, we have an entirely different action from any therapeutical agent known; as compared with mercury, iodine, or the combinations of the iodides, the action of the gold in the combinations named is greater and intensified; that these combinations enter direct into the circulation as gold and arsenic, and spend their force and exert their influence in an alterative way upon the glandular system; that a marked alterative effect is exerted upon all scleroses non-malignant; that it is not only a blood maker, but a blood builder, and a vaso-motor stimulant; that it not only increases the quantity of corpuscles, but the quality of corpuscles; that under its use hæmoglobin is markedly increased; that it is eliminated by the kidneys; that it produces no irritation, either when given per os or hypodermically. I desire to return thanks to Dr. J. E. Cashin and Dr. Purifoy at the Louisville City Hospital, for very valuable assistance.

## Figures and Tables

**FIG. I CASE II. f1:**
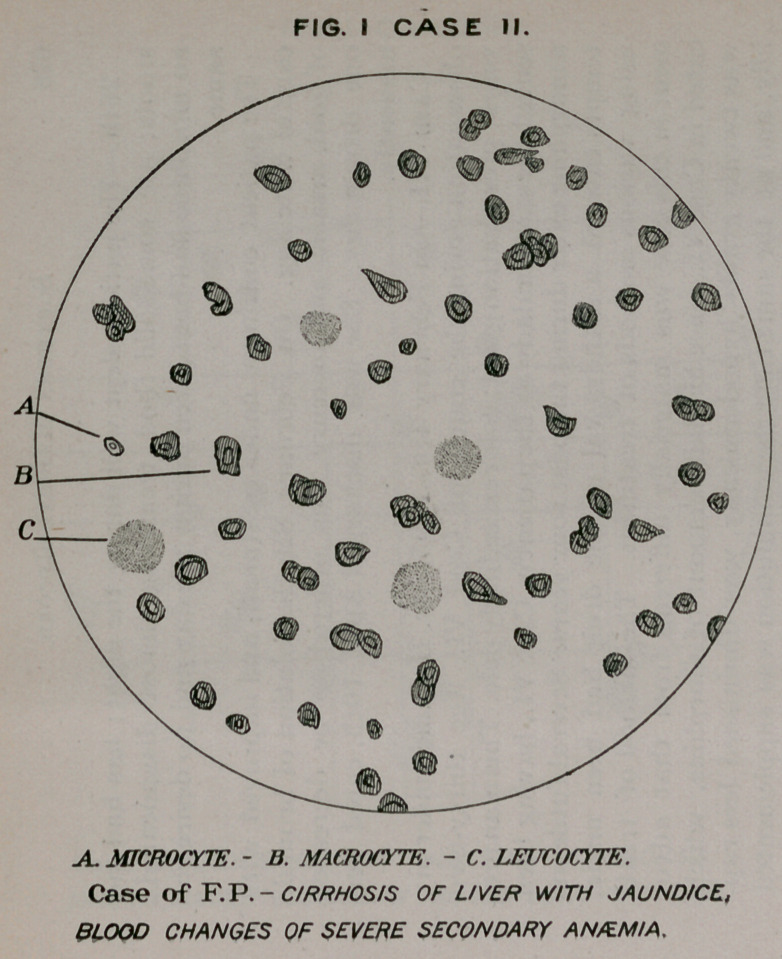


**FIG. II CASE II. f2:**
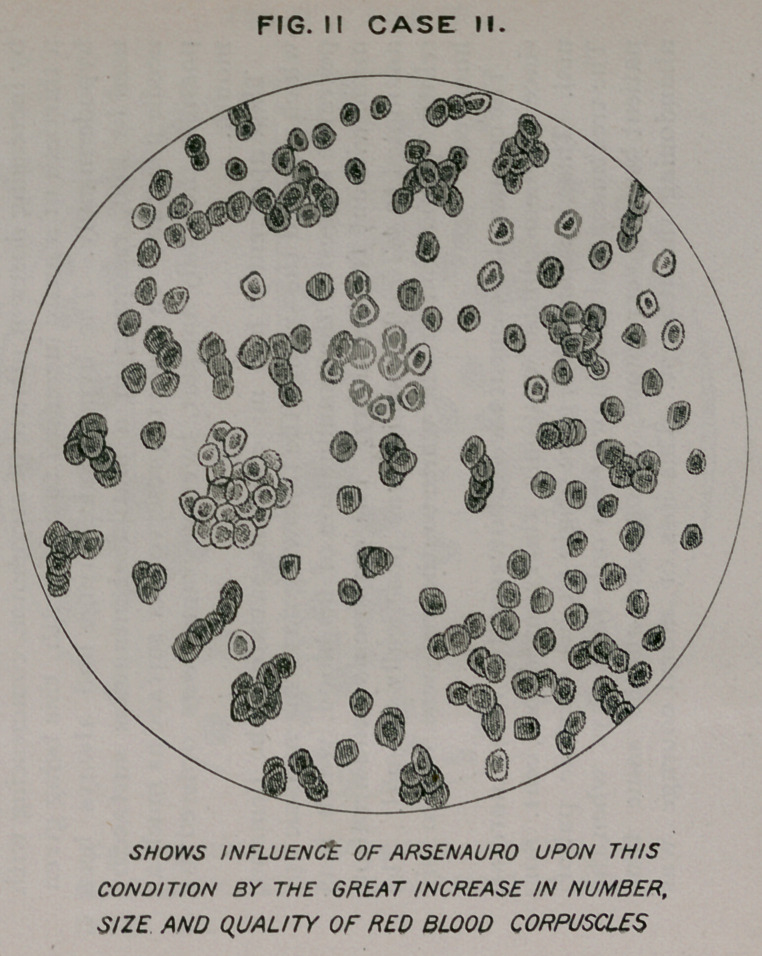


**FIG. I CASE III. f3:**
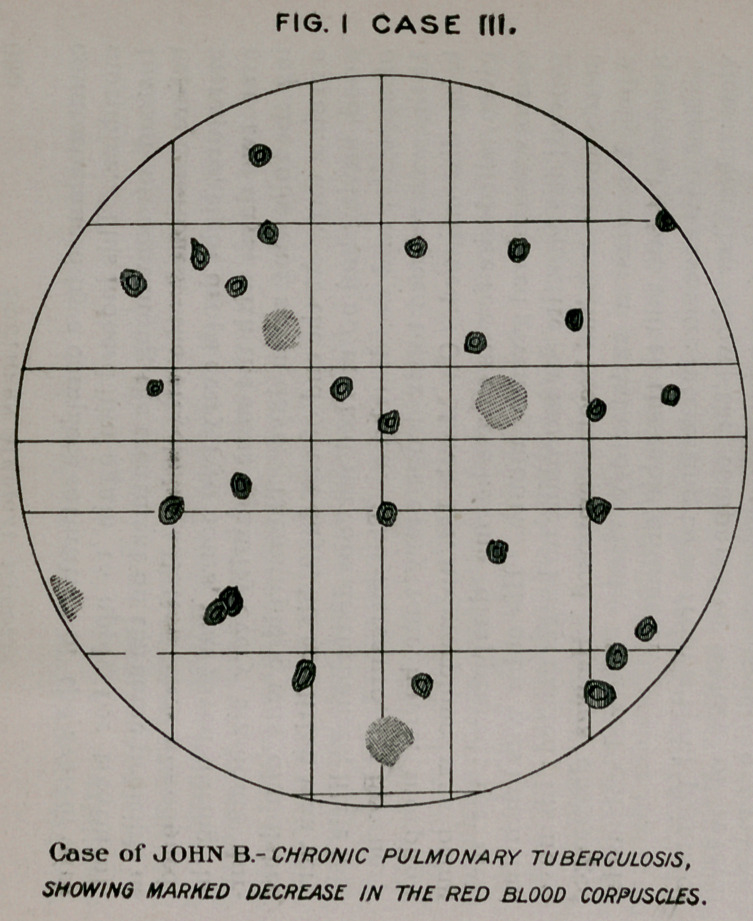


**FIG. II CASE III. f4:**